# Expanding the phenotypic spectrum of *ARID1B*-mediated disorders and identification of altered cell-cycle dynamics due to *ARID1B* haploinsufficiency

**DOI:** 10.1186/1750-1172-9-43

**Published:** 2014-03-27

**Authors:** Joe C H Sim, Susan M White, Elizabeth Fitzpatrick, Gabrielle R Wilson, Greta Gillies, Kate Pope, Hayley S Mountford, Pernille M Torring, Shane McKee, Anneke T Vulto-van Silfhout, Shalini N Jhangiani, Donna M Muzny, Richard J Leventer, Martin B Delatycki, David J Amor, Paul J Lockhart

**Affiliations:** 1 Bruce Lefroy Centre for Genetic Health Research, Murdoch Childrens Research Institute, Parkville, Victoria 3052, Australia; 2 Department of Paediatrics, The University of Melbourne, Parkville, Victoria 3052, Australia; 3 Department of Clinical Genetics, Odense University Hospital, University of Southern Denmark, Odense, Denmark; 4 Northern Ireland Regional Genetics Service, Belfast City Hospital, Belfast, UK; 5 Department of Human Genetics, Radboud University Medical Center, Nijmegen, The Netherlands; 6 Human Genome Sequencing Center, Baylor College of Medicine, Houston, Texas, USA; 7 Department of Neurology, Royal Children’s Hospital, Parkville, Victoria 3052, Australia; 8 Clinical Genetics, Austin Health, Heidelberg, Victoria 3084, Australia

**Keywords:** Intellectual disability, Chromatin remodelling, Coffin-Siris syndrome, ARID1B mutation, Cell cycle, Haploinsufficiency

## Abstract

**Background:**

Mutations in genes encoding components of the Brahma-associated factor (BAF) chromatin remodeling complex have recently been shown to contribute to multiple syndromes characterised by developmental delay and intellectual disability. *ARID1B* mutations have been identified as the predominant cause of Coffin-Siris syndrome and have also been shown to be a frequent cause of nonsyndromic intellectual disability. Here, we investigate the molecular basis of a patient with an overlapping but distinctive phenotype of intellectual disability, plantar fat pads and facial dysmorphism.

**Methods/results:**

High density microarray analysis of the patient demonstrated a heterozygous deletion at 6q25.3, which resulted in the loss of four genes including AT Rich Interactive Domain 1B (*ARID1B*). Subsequent quantitative real-time PCR analysis revealed *ARID1B* haploinsufficiency in the patient. Analysis of both patient-derived and *ARID1B* knockdown fibroblasts after serum starvation demonstrated delayed cell cycle re-entry associated with reduced cell number in the S_1_ phase. Based on the patient’s distinctive phenotype, we ascertained four additional patients and identified heterozygous *de novo ARID1B* frameshift or nonsense mutations in all of them.

**Conclusions:**

This study broadens the spectrum of *ARID1B* associated phenotypes by describing a distinctive phenotype including plantar fat pads but lacking the hypertrichosis or fifth nail hypoplasia associated with Coffin-Siris syndrome. We present the first direct evidence in patient-derived cells that alterations in cell cycle contribute to the underlying pathogenesis of syndromes associated with *ARID1B* haploinsufficiency.

## Introduction

The control of gene expression is an intricately regulated process that requires many multiprotein complexes. Chromain remodeling regulates gene expression by modulating the access of transcription machinery proteins to the condensed genomic DNA via dynamic modification of the chromatin architecture. This modification is mediated by either covalent histone modifications via specific enzymes such as histone acetyltransferases or ATP-dependent alteration of DNA-nucleosome topology [1]. The latter mode of modification is mediated by a class of protein complexes called ATP-dependent chromatin-remodeling complexes, which are known to regulate gene expression in specific cellular contexts or at defined time points [2]. Recent studies have linked mutations in these complexes to both developmental disorders and cancer [3]. Mutations in *ARID1B* and several other genes encoding components of the Brahma-associated factor (BAF, also referred to as switching defective and sucrose nonfermenting SWI/SNF-α) chromatin remodeling complex, were recently shown to cause Coffin-Siris syndrome (CSS) [4,5]. Subsequent studies have demonstrated that mutations in *ARID1B* are the main cause of CSS [6-8]. CSS is characterised by intellectual disability, severe speech impairment, coarse facial features, microcephaly, developmental delay and hypoplastic nails on the fifth digits (MIM 135900). However, *ARID1B* mutations have also been identified in a broader cohort of patients, including nonsyndromic intellectual disability and deletions in individuals with intellectual disability, autism and agenesis of the corpus callosum [9-11]. It is unclear as to why patients with *ARID1B* mutations present such a broad phenotypic spectrum.

*ARID1B* encodes a DNA-binding protein component of the ubiquitous ATP-dependent chromatin remodelling BAF complex, which is known to regulate gene expression, including genes involved in proliferation and differentiation [12]. These complexes are made up of at least ten core proteins and have fifteen known interchangeable components to give rise to an array of functionally distinct and cell-type specific BAF complexes, such as neuronal progenitor BAF complex [12,13]. Thus, mutations in different BAF components are likely to perturb the function of BAF complexes to varied degrees in different cell types. In agreement with this notion, a striking feature of mutations in the BAF components is the phenotypic variability presented by the patients [6,7]. To date, the biological processes affected in patients with *ARID1B* mutations are largely unknown.

Here, we report *ARID1B* haploinsufficiency in five patients with moderate intellectual disability, absent speech and dysmorphic features with narrow palpebral fissures, long eyelashes, a thin upper lip and full lower lip. Alterations in cell cycle were observed in fibroblasts derived from a patient and *ARID1B*-knockdown control fibroblast cells. Our study broadens the phenotypic spectrum of *ARID1B*-mediated disorders and provides the first evidence that alterations in cell cycle contribute to the underlying pathogenesis of syndromes associated with *ARID1B* haploinsufficiency.

## Methods

### Patients

Informed consents were obtained from patients’ parents for the publication of clinical, genetic and molecular analyses. After receiving institutional Ethics Committee (Royal Children’s Hospital, Melbourne, Australia) blood and tissue samples were obtained. Genomic DNA was extracted from whole blood using the BACC DNA extraction kit (GE Healthcare Life Sciences) according to the manufacturer’s instructions. Standard karyotype analysis was performed using G-banding and high density SNP array data (Affymetrix Human SNP array 6.0) was analysed for copy number variation using Karyo-studio (Illumina).

### Generation of *ARID1B* knockdown human fibroblast

Primary fibroblast cultures were established using standard procedures and maintained in BME supplemented with 10% fetal bovine serum (FBS). MicroRNA-adapted shRNA (shRNAmir)-mediated knockdown of *ARID1B* was performed using lentivirus containing *ARID1B*-targeting shRNAmir (V3THS_306691 or V3THS_306692, Open Biosystems) or scrambled non-silencing control (RHS4743, Open Biosystem). The lentiviral particles were generated by transfecting HEK293FT with V3THS_306691, V3THS_306692 or RHS4743 plasmids, and SPAX2 (Addgene) and pMD2.G (Addgene) lentiviral packaging plasmids using Lipofectamine 2000 (Invitrogen) according to manufacturers’ instructions. The viral supernatants were harvested after 48 hours and concentrated at 70, 000 g, 4°C for 2 hours. Wildtype fibroblasts were transduced with both V3THS_306691 (MOI = 20) and V3THS_306692 (MOI = 20) or RHS4743 (MOI = 20) to generate *ARID1B*-knockdown and non-silencing control fibroblast lines respectively. Transduced cells were cultured in complete media containing 4 μg/ml puromycin for 2 weeks and transduction of the fibroblast lines was confirmed by expression of the co-expressed marker protein turboRFP.

### Quantitative real-time PCR analysis

Fibroblast cells were cultured in 60 cm dish and RNA was extracted using SV Total RNA Isolation system (Promega) according to manufacturer’s instructions. 100 ng RNA was used to generate cDNA library for each sample using Transcriptor cDNA First Strand Synthesis kit (Roche) according to manufacturer’s instructions. Real-time PCR reactions were performed in triplicate with Light Cycler 480II instrument (Roche) using Taqman probes directed against *ARID1B* (Applied Biosystems, hs00368175) and the housekeeping control Human Large Ribosomal Protein (*RPLO*) (Applied Biosystems, 432631E-0904009) and Fast Start Taqman Probe Master Mix (Roche) according to manufacturer’s instructions. The relative *ARID1B* expression was analysed using Pfaffl method with respect to *RPLO* expression [14]. Standard deviations were calculated using Gaussian error propagation. T-Test was used to investigate the difference in relative *ARID1B* expression between samples. Each experiment was independently performed at least three times.

### Serum starvation assay

Fibroblasts were cultured in 60 cm dishes and incubated in serum free media for seven days to induce cell cycle arrest. Subsequently, complete media (10% FBS) was added and cells were incubated for up to two days prior to FACS analysis to quantify the number of cells undergoing S_1_ phase. Cells were detached, fixed with 80% ethanol and incubated with propidium iodide/RNAse A staining solution (50 μg propidium iodide/ml, 0.2 mg RNAse A/ml, 0.05% Triton-X100 in PBS) for 2 hours prior to Fluorescence-Activated Cell Sorting (FACS) analysis. A minimum of 10,000 cells were counted for each sample using BD LSR II Flow Cytometer (BD science) with excitation laser set at 488 nm and emission filter set at 695 nm/40 and analysed using ModFit LT analysis software (Verity Software House). Each experiment was independently performed at least three times.

### Sanger sequencing of ARID1B and whole Exome sequencing

Exons of *ARID1B* were amplified via PCR with primer sets listed in Additional file 1: Table S1. The resulting PCR products were purified and sequenced using BigDye Terminator kit v3.1 (Applied Biosystem) and ABI 3730 DNA Analyzer (Applied Biosystems). Exome sequencing of patient 5 was performed on an Illumina Hiseq 2000 platform with the NimbleGen VCRome 2.1 exome capture design for enrichment. The 125 bp paired-end reads were mapped to UCSC genome Browser Hg 19 and variants and indels were called using HGSC Mercury analysis pipeline [15]. High quality variant calls were filtered on nonsynonymous changes in the coding regions or changes affecting the canonical splice sites. Variants that were present with a frequency above 1% in dbSNP and the Nijmegen in-house database containing data of over 1,300 exomes, or in 200 exomes analysed using the same Illumina Hiseq 2000 platform were excluded.

## Results

### Identification of patient 1

**Figure 1:**
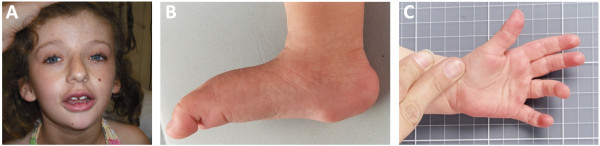
**Clinical phenotype of patient 1.** The facial photographs show dysmorphism including a broad face, narrow palpebral fissures, thin upper lip, full lower lip and low-set ears **(A)**. The plantar fat pads anteromedial to the heel and fetal toe pads are prominent **(B)** and the hands show fetal finger pads and pillowing over the metacarpal heads **(C)**.

**Figure 2:**
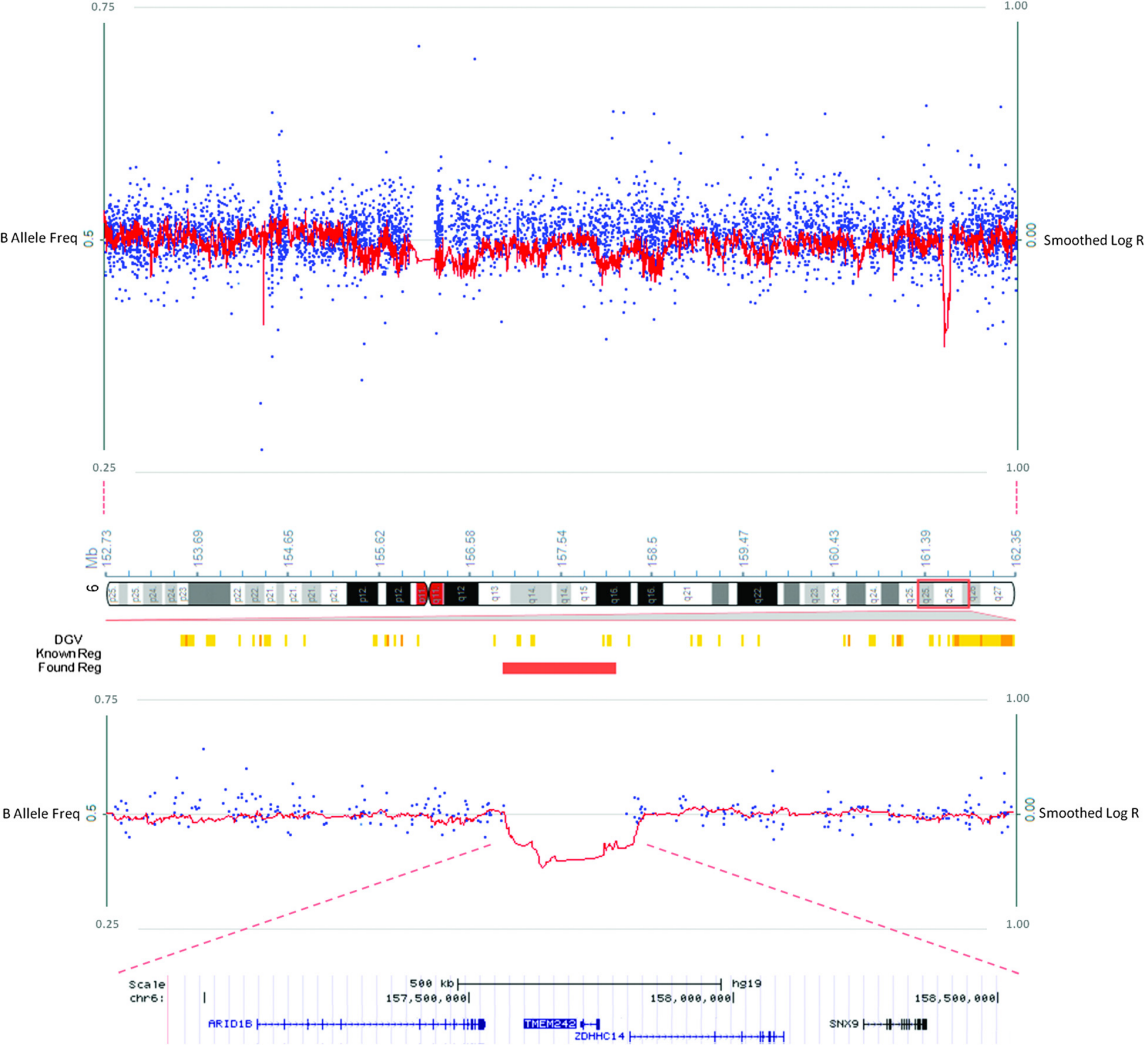
**Molecular characterisation of patient 1.** High density SNP chip array and CNV analysis identified a heterozygous *de novo* 4;6 reciprocal translocation that resulted in the deletion of four genes encoding sorting nexin-9 (SNX9), zinc finger DHHC-type containing 14 (ZDHHC14), transmembrane protein 242 (TMEM242) and AT rich interactive domain 1B (ARID1B) at 6q25.3.

Patient 1 presented with moderate intellectual disability, absent speech and dysmorphic features with narrow palpebral fissures, long eyelashes, a thin upper lip and full lower lip. Fetal finger and toe pads and plantar lipomas were present (Figure 1A-C). Karyotype analysis showed an apparently balanced *de novo* reciprocal translocation involving chromosomes 4 and 6 (46,XY,t(4;6)(q35;q25.3)) in all examined cells (n = 15). The chromosome 4 breakpoint was located within a 0.3 Mb region that did not encode any genes, whereas the chromosome 6 breakpoint was mapped to a ~0.4 Mb region that encoded several genes. Copy number analysis did not identify any variations associated with the chromosome 4 breakpoint. However, a heterozygous deletion of ~1.2 Mb was identified on 6q25.3 that resulted in single allele loss of the genes encoding sorting nexin-9 (SNX9), zinc finger DHHC-type containing 14 (ZDHHC14), transmembrane protein 242 (TMEM242) and AT rich interactive domain 1B (ARID1B) (Figure 2).

### *ARID1B* haploinsufficiency in patient 1

**Figure 3:**
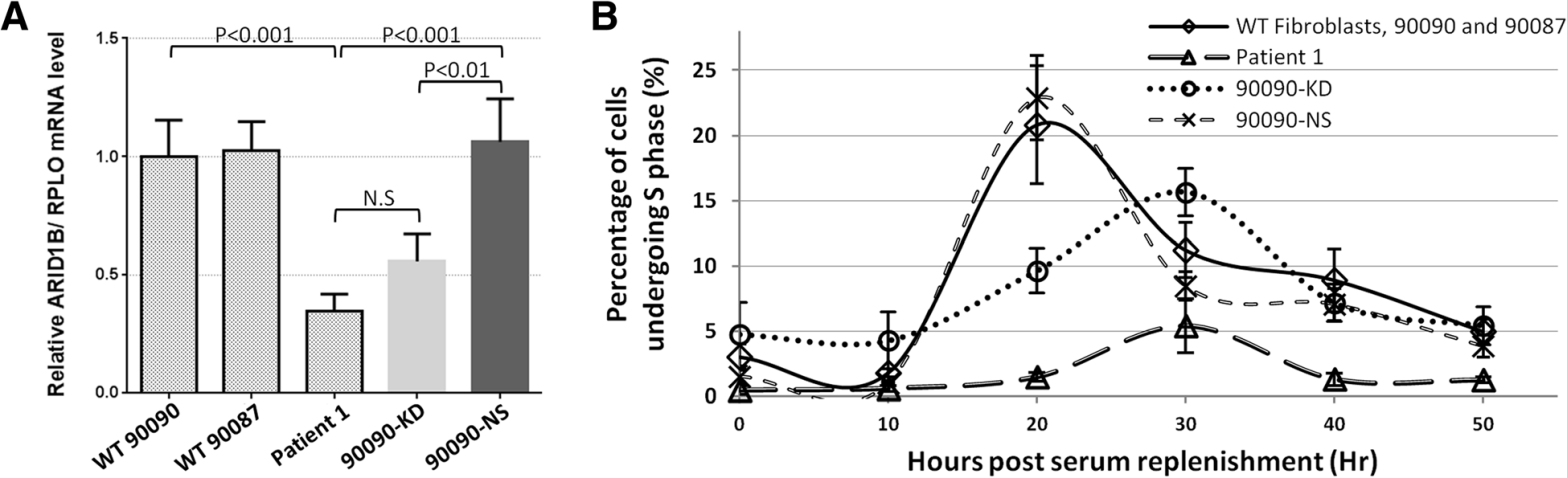
***ARID1B *haploinsufficiency results in delayed S**_**1 **_**Phase entry.** Real Time Quantitative PCR analysis of patient-derived (Patient 1) and control (90087 and 90090) fibroblasts was performed using Taqman probes directed against *ARID1B* and the housekeeping control *RPLO*. Samples were normalised to control 90090, which was assigned a value of 1 and analysed by the Pfaffl relative quantification method. The expression of *ARID1B* was significantly reduced in both Patient 1 and the control 90090 after shRNAmir mediated knockdown of *ARID1B* (90090-KD), whereas the non-silencing control did not alter *ARID1B* expression (90090-NS) **(A)**. Cell cycle dynamics of serum-starved cells undergoing S_1_ phase after serum replenishment was analysed using ModFit LT. The percentage of cells undergoing S_1_ phase at 20 hours post serum replenishment was significantly lower in both patient 1 (P = 0.0002) and 90090-KD (P = 0.005) compared to control **(B)**.

Given the documented role of *ARID1B* in disorders with a clinical presentation somewhat similar to our patient, we tested the effect of the deletion on *ARID1B* expression in patient-derived primary fibroblasts. Western blot analysis using multiple commercially available antibodies directed against ARID1B failed to robustly identify the predicted ~240 kDa protein in patient or control fibroblast cells (data not shown). However, real-time PCR analysis revealed that the transcription level of *ARID1B* in patient 1 was significantly reduced (35 ± 7%, mean ± SD, n = 5, P < 0.001) in comparison to the control fibroblasts (Figure 3A). In order to further characterise the effect of *ARID1B* haploinsufficiency, we utilised a knockdown approach. Wildtype fibroblasts co-transduced with *ARID1B*-targetting shRNAmir (V3THS_306691 and V3THS_306692) expressed *ARID1B* mRNA at approximately 60% of control levels (56 ± 12%, mean ± SD, n = 3, P < 0.01). In contrast, no effect on *ARID1B* mRNA level was observed when the non-silencing control was utilised (Figure 3A).

### Entry to S_1_ phase is delayed in patient 1

It was previously demonstrated that ARID1B deficiency delayed cell cycle re-entry after cell cycle arrest induced by serum starvation in mouse MC3T3 osteoblasts [16]. To investigate whether the molecular defect in patient 1 might lead to similar deficits, we analysed the kinetics of cell cycle re-entry after serum starvation [17]. Control fibroblasts showed a peak in cells undergoing S_1_ phase (21% ± 4.5, mean ± SD, n = 6) at 20 hours post serum replenishment (Figure 3B). In contrast, the percentage of cells undergoing S_1_ phase at 20 hours post serum replenishment was significantly lower in both patient 1 (1.5% ± 0.35, mean ± SD, n = 3, P = 0.0002) and 90090-KD (9.6% ± 1.7, mean ± SD, n = 3, P = 0.005) compared to control. The maximum number of patient 1 cells in S_1_ phase (5.4 ± 2.1%, mean ± SD, n = 3) was observed 30 hours post serum replenishment. Similarly, knockdown of *ARID1B* in control fibroblasts resulted in a delayed entry to S_1_ phase, with the peak also occurring at 30 hours post serum replenishment. The number of cells undergoing S_1_ phase in the knockdown fibroblasts at 30 hours post serum replenishment was greater than observed in patient 1. This may be because there was a trend to elevated *ARID1B* mRNA (and presumably protein) compared to patient 1, although it was not of statistical significance (Figure 3A). Both patient 1 and the knockdown cells demonstrated significantly reduced mRNA levels compared to the control fibroblasts and control fibroblasts transduced with the non-silencing ‘scramble’ control. While off-target effects were not specifically controlled for, the non-silencing control cells did not display alterations in either the number or timing of cells undergoing S_1_ phase. Moreover, both patient and ARID1B-knockdown cells displayed similar delayed entry to S_1_ phase, suggesting the effects were due to reduced ARID1B rather than a non-specific off target effect of the *ARID1B*-targetting shRNAmir used. Although we could not definitively demonstrate reduced endogenous ARID1B levels by western blot (antibodies tested were sc-32762 and NB100-57484), collectively these observations suggested that reduced ARID1B was associated with perturbed cell cycle regulation, which affected both the number and timing of cells entering S_1_ phase after serum starvation.

### Identification of four additional patients with *ARID1B* mutations

**Figure 4:**
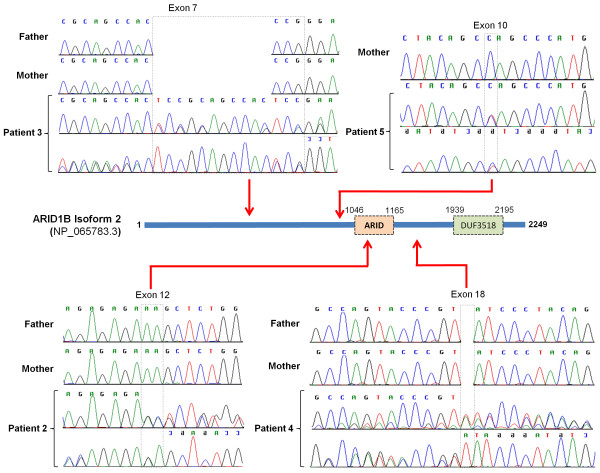
**Analysis of *ARID1B *in the patient cohort.** The protein domain organisation of ARID1B and the position of the four mutations identified by sequencing are shown. The forward and reverse sequencing traces demonstrate the NM_020732.3(ARID1B):c.3208_3209delAA (p.(Lys1070Alafs*47)) mutation in patient 2 (A), the NM_020732.3(ARID1B):c.2306_2308delCCGinsTCCGCAGCCACTCC (p.(Pro769Leufs*17)) mutation in patient 3 (B), the NM_020732.3(ARID1B):c.4273dupT (p.(Tyr1425Leufs*34)) mutation in patient 4 (C) and the NM_020732.3(ARID1B):c.2941C > T (p.(Gln981*)) mutation in patient 5 (D). Where available, the forward sequencing trace for the parents is also shown. Mutation co-ordinates are derived from refseq NM_020732.3.

The distinctive clinical features observed in patient 1 enabled us to ascertain four additional patients with a strikingly similar phenotype. All five patients, including patient 1 had intellectual disability, absent speech, plantar fat pads, fetal finger pads, and facial dysmorphism, consisting of narrow palpebral fissures, long eyelashes, a thin upper lip and full lower lip (Table 1). Direct sequencing of genomic DNA revealed that three additional patients encoded *de novo* single allelic *ARID1B* mutations; NM_020732.3(ARID1B):c.3208_3209delAA (p.(Lys1070Alafs*47) in patient 2, NM_020732.3(ARID1B):c.2306_2308delCCGinsTCCGCAGCCACTCC (p.(Pro769Leufs*17)) in patient 3 and NM_020732.3(ARID1B):c.4273dupT (p.(Tyr1425Leufs*34)) in patient 4. Whole exome sequencing of patient 5 identified 325 unique variants. Further filtering against an in-house database of genes previously implicated in intellectual disability identified a single candidate variant, which is predicted to be truncating. The *de novo* heterozygous *ARID1B* mutation NM_020732.3(ARID1B):c.2941C > T (p.(Gln981*)) was confirmed by Sanger sequencing (Figure 4). Two of these patients (patient 3 and patient 5) were previously published as having a phenotype consistent with Pierpont syndrome [18]. The molecular basis of Pierpont syndrome remains to be reported, but plantar fat pads are a key feature. The facial features in the five patients described here are distinct from those originally reported in Pierpont syndrome in that their palpebral fissures are not as narrow and their nasal tip not as broad [18,19]. Therefore we now believe their diagnosis should be of a BAF complex disorder due to *ARID1B* haploinsufficiency. In addition, we carried out mutation analysis of *ARID1B* (but not other members of the BAF complex) in four patients with classical features of Pierpont syndrome including patient 1 of the original case report [19] and did not identify any mutation (unpublished data).

**Table 1:** Comparison of patient clinical features

	**Patient 1**	**Patient 2**	**Patient 3**	**Patient 4**	**Patient 5**
Previously published	-	-	Patient 5 in Wright et al., 2011 [18]	-	Patient 6 in Wright et al., 2011 [18]
Age at assessment	8y	2y	6y 10m	5y	8y 2m
Height centile	50th	10-25th	25th	3rd	2nd
Weight centile	50-75th	25th	50-75th	25th	2nd
Head circumference	50-98th	25th	50-98th	25th	50th
Heel Fat pads	+	+	+	+	+
Fetal finger and toe pads	+	+	+	NR	+
Fifth nail hypoplasia	-	-	-	-	-
Hirsutism	-	-	-	-	+
ID	Mod	Mod	Mild	Mod	Mod
Speech	Absent	Absent	Delayed	Absent	Delayed
Seizures	-	+	-	+	-
Drooling	+	+	+	-	+
Feeding difficulty	+	-	Mild	-	-
Scoliosis	-	-	-	-	+
Inguinal hernia	-	-	-	-	-
Neuroimaging	Mega cisterna magna on brain MRI	Normal brain MRI	Hypoplastic posterior elements of corpus callosum on MRI	Head CT in first year of life, normal	Normal

## Discussion

Recent reports have demonstrated that mutations in *ARID1B* can cause both nonsyndromic intellectual disability and CSS. The phenotypes associated with *ARID1B* haploinsufficiency are variable but common features of the syndromic and nonsyndromic cases include intellectual disability and speech impairment. While the facial dysmorphism and intellectual disability present in our cohort is similar to previously published patients with *ARID1B* mutations [4-7], this study broadens the spectrum of *ARID1B*-associated phenotypes because all our patients presented with plantar fat pads and fetal finger/toe pads and none of our patients have the fifth nail hypoplasia hallmark of CSS. As some of the previous cohorts with *ARID1B* mutations were ascertained by a clinical diagnosis of CSS, there is a selection bias in the phenotypic features they will have. Although none of the point mutations in *ARID1B* are common between CSS and our cohort, three CSS-like patients with *de novo* single allelic deletion at chromosome 6 similar to patient 1 were identified by Santen *et al.* in a large cohort of patients with intellectual disability [4]. The phenotypes of these patients share some features but apparently represent distinct syndromic manifestations of *ARID1B* haploinsufficiency. Thus, our cohort demonstrates that the *ARID1B* phenotypic spectrum is broader than previously defined and includes the novel clinical features of plantar fat pads and fetal digit pads. The constant features across cohorts appear to be the facial gestalt and the presence of intellectual disability particularly affecting speech, in contrast to the marked variability in the presence and type of manifestations in the hands and feet.

ARID1B is a DNA-binding component of BAF complexes that are involved in regulating many biological pathways [12]. However, the repertoire of pathways regulated by these BAF complexes and the impact of mutations in BAF components on these pathways are still poorly understood. Here, we present the first direct evidence in patient-derived cells that alterations in cell cycle may contribute to the pathogenesis of syndromes associated with *ARID1B* haploinsufficiency. Given that the BAF complex comprises over 25 core and interchangeable protein subunits that give rise to functionally distinct and cell-type specific complexes [12], it is likely that additional variation in these components contributes to the observed phenotypic variability in ARID1B-mediated disorders.

It is possible that the developmental features observed in patients with *ARID1B* haploinsufficiency are the result of abnormal regulation of cell cycle re-entry of developmentally arrested cells. This would impair developmental processes by upsetting the initiation of progenitor cell proliferation. In support of this notion, homozygous knockout of *arid1b* in mouse is embryonic lethal but the ES cells demonstrated a reduced proliferation rate and perturbation of differentiation and cell cycle [20]. An analogous observation is the presence of microcephaly in a minority of patients with *ARID1B* mutations and more broadly in Coffin-Siris syndrome [6,8]. Besides regulating cell cycle, chromatin-remodeling events and BAF complexes have been shown to be critically important during neural development and dendrite formation [13,21,22]. Although a specific role for ARID1B in early brain development remains to be demonstrated, the gene is predominantly expressed in neural tissues in the developing mouse embryo, suggesting that it is important for development of the brain when multipotent neuroepithelial cells are actively proliferating [9,23]. Future studies should investigate if impaired neural development contributes to the intellectual disability and speech impairment that are consistently observed in patients with *ARID1B* haploinsufficiency.

## Conclusion

Our study demonstrates that the *ARID1B* phenotypic spectrum is broader than previously defined and includes the novel clinical features of plantar fat pads and fetal digit pads. These features can manifest in the absence of the hypertrichosis or fifth nail hypoplasia associated with Coffin-Siris syndrome. In addition, we present the first direct evidence in patient-derived cells that alterations in cell cycle may contribute to the underlying pathogenesis of syndromes associated with *ARID1B* haploinsufficiency.

## Competing interests

The authors declare that they have no competing interests.

## Authors’ contributions

JS performed molecular analysis and co-wrote the manuscript. SW provided and interpreted the clinical data and co-wrote the manuscript. EF, GW, GG, HM, SJ and DM performed molecular analysis and interpreted data. KP performed patient recruitment and read/contributed to the manuscript. PT, ATVvS, SM, RJL, MBD and DJA provided and interpreted the clinical data and revised the manuscript. PJL collected and analysed the data, wrote the manuscript and is responsible for overall content as guarantor and corresponding author.

## Supplementary Material

Additional file 1: Table S1 Details of primer sequences. Primers were designed to amplify DNA encoding *ARID1B* (NM_020732.3) for direct sequence analysis. Click here for file
