# Supplementary material for: Expanding the phenotypic spectrum of ARID1B-mediated disorders and identification of altered cell-cycle dynamics due to ARID1B haploinsufficiency
Source: Orphanet J Rare Dis. 2014 Mar 27;9:43. doi: 10.1186/1750-1172-9-43 (PMC4022252; doi:10.1186/1750-1172-9-43)
Supplement: Additional file 1: Table S1 — Details of primer sequences. Primers were designed to amplify DNA encoding ARID1B (NM_020732.3) for direct sequence analysis. [file 1750-1172-9-43-S1.docx]

**Table S1: Details of primer sequences.**

Primers were designed to amplify DNA encoding *ARID1B* (NM_020732.3) for direct sequence analysis.

| ***Primer*** | **sequence** |
| --- | --- |
| ***hARID1B-5'UTR-1F*** | **GCTCGTCAGACTCGTTTTCC** |
| ***hARID1B-5'UTR-1R*** | **GGGAGACAATAACCCCCACT** |
| ***hARID1B-5'UTR-2F*** | **ACCATGAAAGCACACAGCAG** |
| ***hARID1B-5'UTR-2R*** | **GAGGAGGAGGAGGAGGACAG** |
| ***hARID1Be1-1F*** | **CCCCGTCACGAACTCAAC** |
| ***hARID1Be1-1R*** | **CTTTGTTGTCCGCCATGTT** |
| ***hARID1Be1-2F*** | **GCCGTCCCGGAGTTTAATA** |
| ***hARID1Be1-2R*** | **AGGTGAGCAGCTGATTGAGG** |
| ***hARID1Be1-3F*** | **GCAGGAGCAGGAGGAGCA** |
| ***hARID1Be1-3R*** | **AGTACGCGGCAAAGAAAGAA** |
| ***hARID1Be2F*** | **TGCTTATGGTCCAAAATCGTT** |
| ***hARID1Be2R*** | **GGACAGCAAAAGGCAAAGAC** |
| ***hARID1Be3F*** | **CCTGGCATCCAGTAGGTGAT** |
| ***hARID1Be3R*** | **CCCTGGAAGGACCTGGTATC** |
| ***hARID1Be4F*** | **AGAAAGCAGCGTGTCGATTT** |
| ***hARID1Be4R*** | **TGACAGGGAACAAGCAATGA** |
| ***hARID1Be5F*** | **GATCTGCCTGCCTTGATCTC** |
| ***hARID1Be5R*** | **CACCTGCACACAAAATCGAC** |
| ***hARID1Be6F*** | **TGGGTGTTACCCAGAAAACC** |
| ***hARID1Be6R*** | **TCTCGTATGCCTTGGTGAGA** |
| ***hARID1Be7F*** | **GCCCAGCTCTCCTACGTTTT** |
| ***hARID1Be7R*** | **AAAGCATCCTCCCTCCTCTC** |
| ***hARID1Be8F*** | **TCGGTCACTGTTGCTTTTTG** |
| ***hARID1Be8R*** | **TCTACAACTTGCTGCCATGC** |
| ***hARID1Be9F*** | **GCAACAGGAAGGGCCTATAA** |
| ***hARID1Be9R*** | **CCAGGGAACAATGCAGCTAT** |
| ***hARID1Be10F*** | **GGGCAAACCACTGCTAATGT** |
| ***hARID1Be10R*** | **CGAAATGTTTGTCTCCACCA** |
| ***hARID1Be11F*** | **GGTGCTGTTTGCATTTCAGA** |
| ***hARID1Be11R*** | **ATGGCTGCAAGTACCGAAAC** |
| ***hARID1Be12F*** | **CCTTCTTCCCTCTCCCTCTG** |
| ***hARID1Be12R*** | **TCTAGTCTGGCCCGCTGTAT** |
| ***hARID1Be13F*** | **CTCCGAGCAGTGCTTTTTCT** |
| ***hARID1Be13R*** | **CTGTTGGCCTGACTGTGAAA** |
| ***hARID1Be14F*** | **TCTTCTGCCTAAAGCCTCCTT** |
| ***hARID1Be14R*** | **GTGAAGTTTGCCCCAGAAAA** |
| ***hARID1Be15F*** | **GGTTTTTGCATTTGCACATTT** |
| ***hARID1Be15R*** | **CTTGGCACGTGTAAGGAGGT** |
| ***hARID1Be16F*** | **GGTGGTGGATGCCTGTAATC** |
| ***hARID1Be16R*** | **TTTCACAGGAAAGGGGACAC** |
| ***hARID1Be17F*** | **CATTCCCAAAATGGGTGTTC** |
| ***hARID1Be17R*** | **ATGTGTTTCAAGAAACCGTGT** |
| ***hARID1Be18-1F*** | **GTGGCACAGCCTAAGGAGAG** |
| ***hARID1Be18-1R*** | **AAGGCATATCATTGCGTGCT** |
| ***hARID1Be18-2F*** | **GAATCCCGCCTCAGATGAT** |
| ***hARID1Be18-2R*** | **TGAGCAGGAACAGCATCTTT** |
| ***hARID1Be19F*** | **AGGACCATCTGTGCTCAACC** |
| ***hARID1Be19R*** | **ACAGTGTTGGACGGTCAGTG** |
| ***hARID1Be20-1F*** | **GGTATTGACGGGTCTCAGGA** |
| ***hARID1Be20-1R*** | **AGGAGGAATTTCCATCTTGC** |
| ***hARID1Be20-2F*** | **GCGTGTGCAGGAGTTCAATA** |
| ***hARID1Be20-2R*** | **CTCATAGGTCTGCGGTGCTC** |
| ***hARID1Be20-3F*** | **ATCCTGGGGAAGCTGATTCT** |
| ***hARID1Be20-3R*** | **CTTGGGCAAGGTTCGATAAA** |
| ***hARID1Be20-4F*** | **GCCACTCCTCCATTTAGTCG** |
| ***hARID1Be20-4R*** | **TAGTGAATGGGGCAGAGGAG** |
| ***hARID1B-3'UTR-1F*** | **GAGAAGGCAAGCATGTGTGA** |
| ***hARID1B-3'UTR-1R*** | **CCTCCTTGAGCCCCTATACC** |
| ***hARID1B-3'UTR-2F*** | **CATCCCATCACCCAAAGTTC** |
| ***hARID1B-3'UTR-2R*** | **TGATCAGCTGCTCTCCTGAA** |
